# Use of Table Sugar and Artificial Sweeteners in Brazil: National Dietary Survey 2008–2009

**DOI:** 10.3390/nu10030295

**Published:** 2018-03-01

**Authors:** Luana Silva Monteiro, Bruna Kulik Hassan, Paulo Rogério Melo Rodrigues, Edna Massae Yokoo, Rosely Sichieri, Rosangela Alves Pereira

**Affiliations:** 1 Curso de Nutrição, Universidade Federal do Rio de Janeiro, Campus Macaé, Av. Aluizio da Silva Gomes, 50-Novo Cavaleiros, CEP 27930-560 Macaé, RJ, Brazil; luananutrir@gmail.com; 2 Instituto de Medicina Social, Universidade do Estado do Rio de Janeiro, Rua São Francisco Xavier, 524, Pavilhão João Lyra Filho, 7º andar, CEP 20550-900 Rio de Janeiro, RJ, Brazil; bkulik@gmail.com (B.K.H.); rosely.sichieri@gmail.com (R.S.); 3 Faculdade de Nutrição, Universidade Federal de Mato Grosso, Avenida Fernando Corrêa da Costa, 2367, Bairro Boa Esperança, CEP 78060-900 Cuiabá, MT, Brazil; prmr84@gmail.com; 4 Departamento de Epidemiologia e Bioestatística, Universidade Federal Fluminense, Rua Marques de Paraná, 303, 3º andar, Centro, CEP 24030-210 Niterói, RJ, Brazil; eyokoo@gmail.com; 5 Departamento de Nutrição Social e Aplicada, Universidade Federal do Rio de Janeiro, Avenida Carlos Chagas Filho, 373, Edifício do Centro de Ciências da Saúde, Bloco J, 2º andar, Cidade Universitária, CEP 21941-590 Rio de Janeiro, RJ, Brazil

**Keywords:** Keywords: sugar, artificial sweeteners, survey, food consumption, energy intake

## Abstract

The study aimed to describe the use of table sugar and artificial sweeteners (AS) in Brazil. A representative sample (*n =* 32,749) of individuals aged > 10 years was examined from the Brazilian National Dietary Survey (2008–2009). Participants reported whether they use table sugar, AS, both, or none as sweeteners for their foods and beverages. Energy intake and the contribution of selected food groups to energy intake were evaluated according to the type of sweetener reported. Sample weights and design effects were considered in the analysis. The majority of the population (85.7%) used sugar to sweeten foods and beverages, 7.6% used AS, and 5.1% utilized both products. The use of AS was more frequent among the elderly (20%), women (10% versus 5.5%), overweight individuals (10% versus 6%), those who live in urban areas (8.5% versus 3%), and those who belong to the highest income quartile (14% versus 1.6%), compared with men, normal weight individuals, those who live in rural areas, and those who belong to the first income quartile, respectively. Overall, the mean daily energy intake of individuals using only sugar was approximately 16% higher than those who used AS exclusively. The contribution of staple foods to daily energy intake was higher in individuals who used sugar than those who used AS.

## 1. Introduction

The consumption of sugars and artificial sweetener (AS) and their effects on health have been widely studied [1,2]. However, information on the use of these products is still limited at national levels, as shown in a systematic review that revealed that only nine countries evaluated the intake of sweeteners in nationally representative dietary studies [3].

The most common sugars in our diet are monosaccharides (glucose, fructose, and galactose) and disaccharides (lactose, maltose, and sucrose), and a major source of dietary sugar is table sugar from drinks and other foods. Table sugar consists mainly of sucrose, and is obtained by industrial processes, usually from sugar cane (*Saccharum officinarum* L.) or from beet (*Beta alba* L.) [4].

According to Popkin and Nielsen [5], the use of sugars has increased worldwide. Analyzing data from 103 countries, the authors conclude that between 1962 and 2000, the intake of caloric sweeteners increased by 74 kcal a day, which means a 15% increase in contribution of these products to total caloric intake (from 9.5% to 10.9%). In Brazil, the first National Dietary Survey, which was conducted in 2008–2009, indicated that on average, total sugars represented 11.4% of the total energy intake of individuals aged >10 years [6], and that 61% of the population consumed more than >10% free sugars for their total energy intake [7], which is the limit recommended by the World Health Organization (WHO) [8].

The harmful consequences of sugars on health were pointed out by Lustig et al. [9], including the development of metabolic syndrome components such as hypertension, high serum triglycerides, and insulin resistance, and alterations on ghrelin, leptin, and dopamine, compelling individuals to eat more. Moreover, sugars can accelerate the aging process by damaging deoxyribonucleic acid (DNA), and specifically fructose has been known to alter the liver function similarly to alcohol [9]. In addition, according to the WHO, increased sugar intake may be related to unhealthy diet and weight gain. Another concern is the association between sugar intake and dental caries [8].

Parallel to the increase of sugar consumption, in recent years, the use of AS has also increased [10]. AS are naturally occurring or artificially generated substances that have a higher sweetening capacity than sugar, and zero or close to zero caloric content [11]. Some of the most commonly used AS are saccharin, cyclamate, aspartame, acesulfame, stevioside, and sucralose. These sweeteners are used in many products, mostly beverages, yogurts, candies, desserts, and gum; frequently, a blend of AS or a blend of sugar plus AS are used to make products’ flavor more acceptable [2].

Thus, artificial sweeteners emerged as an alternative for diabetics; however, they became commonly used for weight loss along with the growth of diet programs for weight loss in the American population, especially women. In the 1950s, the advertising of sweeteners manufacturers and industrial production of food and beverages associated with these products also increased [10]. In Brazil, information on the evolution of AS consumption and its use is limited. However, it is possible that the same process that is occurring in countries such as the United States [12,13,14] is also present in this country.

A population-based study evaluated the use of AS in adults (>20 years) in a city in southern Brazil, and revealed that the prevalence of AS use was 19%, which was almost four times higher among the elderly (≥60 years old) than among 20–29-year-old individuals (32.0% versus 8.7%) [15]. In the United States, Mattes and Popkin [16] examined the food and beverage consumption with AS from 1965 to 2004, and observed that the proportion of individuals (≥2 years) who consumed at least one product with AS increased from 3% in 1965 to 15% in 2003–2004. These authors observed that the increase in AS products was not followed by a decrease in beverages and foods added with sugar. This fact addresses the question on the compensatory energy intake related to AS, either because one feels that a low-calorie beverage or food enables eating more calories, or due to changes in the appetite/hunger control [2]. The adverse contributions of AS on health have also been the focus of a number of studies; however, according to Gardner et al. [2], the results are not sufficient to conclude whether AS can be considered as a beneficial factor on appetite, energy balance, body weight, or cardiometabolic risk factors.

The standard recommendations on the use of AS have not been established yet; however, there are indications on intake levels that are considered safe, which varies depending on the type of AS [17]. Nevertheless, the American Academy of Nutrition and Dietetics adopts the position that the use of AS, as well as sugars, is safe so long as consumers maintain an eating plan that is consistent with the Dietary Guidelines for Americans [18]. However, most of the Americans do not follow such an eating pattern, but commonly follow a Western diet, in which the use of AS to substitute sugar may not be a healthy option [19].

Considering the relevance of characterizing and monitoring the consumption of sugars and AS, the present study aimed to evaluate the use of table sugar and AS in the Brazilian population, and evaluate the variations in daily energy intake and diet composition according to the type of sweetener added in beverages and food. Additionally, a detailed characterization of this consumption in the population (according to sex, age group, weight status, geographical region, and income) contributes to understand Brazilians’ behavior in sweetening foods and beverages.

## 2. Materials and Methods

We used data from the first Brazilian National Dietary Survey, which was conducted along with the 2008–2009 Household Budget Survey developed by the Brazilian Institute of Geography and Statistics (in Portuguese: *Instituto Brasileiro de Geografia e Estatística*). The dietary survey was conducted in a subsample of households included in the budget survey, which comprised a total of 34,003 individuals aged ≥ 10 years [7]. In this study, pregnant and lactating women were excluded (*n* = 1254) because they might have temporarily altered their eating habits. Therefore, a total of 32,749 individuals were analyzed.

To identify the type of sweetener consumed by the Brazilian population, we used a separate question from the Brazilian National Dietary Survey about sugar and AS consumption. Individuals were asked what sweetener they most commonly used to sweeten foods (solids or liquids), using the following four answer options: “sugar”, “AS”, “sugar and AS”, or “none of these two substances”, The question asked to the participants was "What do you often use to sweeten the foods (solids or liquids) you consume: “sugar”, “artificial sweeteners”, “sugar and artificial sweeteners”, or “do not use either” [7].

Dietary intake was evaluated during the 12-month research period in a way that all of the geographic and socioeconomic strata were considered in the four quarters. Dietary intake was estimated based on dietary records, and individuals were instructed to record all of the foods and beverages (except water) that they consumed in two non-consecutive days, including the amount (portion sizes or volume measurements), time, and place (at home or away from home) [7]. In this study, data from the first day of the food record was analyzed.

Energy intake was estimated based on the food composition database compiled for the Brazilian dietary survey [20]. The foods cited in the food records were categorized into 58 food groups according to their nutritional characteristics and use in the diet [6]. In order to assess the differences in food intake according to the product used to sweeten food, only the food subsets reported by at least 10% of the population (20 subgroups) were considered.

The prevalence of using table sweetener (table sugar, AS, sugar and AS, none of these two substances) was estimated according to the total population and in the following strata: sex, age group (adolescents, 10–19 years; adults, 20–59 years; and elderly, ≥60 years), macroregions (north, northeast, southeast, south, and midwest), urban or rural areas, per capita family income (estimated from the total household income divided by the number of household members) categorized into quartiles, and weight status evaluated using body mass index (BMI = weight/height^2^), using the WHO criteria (adolescents were classified as overweight if *z*-scores of BMI were >+1 of the reference distribution [21]; adults were overweight if the BMI was ≥25 kg/m^2^ [22]); and elderly were considered overweight if the BMI was ≥27 kg/m^2^ [23].

The variations in energy intake and caloric contribution (%) of food groups to daily energy intake according to the use of sweetener among the population strata were estimated using generalized linear models (GLM) developed in the Complex Sample module (SPSS, IBM, Armonk, NY, USA), with the Bonferroni correction when comparing the products used to sweeten foods and beverages. Homogeneity in the categorical variables according to the population strata were evaluated using the chi-square test. Sample weights and design effects were considered in the analysis. Data were analyzed using the Statistical Package for Social Sciences (SPSS, version 19, IBM, Armonk, NY, USA).

The research protocol was approved by the Ethics Committee of the Institute of Social Medicine at the State University of Rio de Janeiro (CAAE 0011.0.259.000-11) on 19 July 2011. All subjects gave their informed consent for inclusion before they participated in the study. The study was conducted in accordance with the Declaration of Helsinki.

## 3. Results

In this study, 64.6% of the sample were adults, 49.9% were females, and 43.2% lived in the southeastern region. The proportion of overweight or obese individuals reached 41.8%. Overall, the most commonly used product to sweeten foods and beverages was sugar, which was reported by 85.7% of the population, with 5.1% using both sugar and AS. The use of sugar decreased with age, and was slightly more frequent in men than in women (89.4% versus 82.0%), and in rural than in urban areas (93.3% versus 84.2%). The exclusive use of sugar decreased as the per capita income increased, as observed in 96.0% of individuals belonging to families in the lowest income quartile compared to 74.1% in the highest income quartile. The use of sugar was less frequent in the south and southeast regions than in other regions (Table 1).

The exclusive use of AS was reported by 7.6% of the population, and in contrast with the use of sugar, it increased with age (adolescent, 1.9%; adult, 6.9%; elderly, 19.9%); was more frequent in women compared with men (10.0% versus 5.5%), overweight/obese individuals compared with those with normal weight (10% versus 6%), urban compared with rural areas (8.5% versus 3%); and was three times less common in the north (2.9%) than in the south and southeast (9.5% in both) areas, and nine times lower in individuals belonging to the first quartile of income (1.6%) compared with the highest quartile (14.0%) (Table 1).

In all of the population strata analyzed, the mean daily energy intake of individuals who used sugar only was approximately 16% higher than those who used AS only, except in the midwest. In this region, no difference was observed in the mean daily energy intake between individuals who used sugar or AS. Among those who reported using both sugar and AS, the average daily energy intake was approximately 6% lower than those who used sugar only (1832 kcal versus 1955 kcal). The average daily energy intake of those who reported not using sugar or AS (1770 kcal) was 10% lower than those who used sugar only (Table 2).

The contribution to daily energy intake was higher for individuals who reported using sugar when compared with those who used AS for rice (14.9% versus 12.2%), beans (10.7% versus 7.8%), coffee and tea (6.9% versus 2.6%), fruit juices (4.8% versus 3.5%), sweets and desserts (5.0% versus 4.0%), roots and tubers (3.9% versus 3.3%), sugar-sweetened beverages (2.3% versus 1.6%), and eggs (1.5% versus 0.7%). On the other hand, individuals who used sugar had a lower contribution to daily energy intake when compared with those who used AS for fruit (2.7% versus 5.5%), bread (8.6% versus 10.8%), cheese (0.9% versus 2.5%), poultry (4.7% versus 6.2%), snacks and chips (1.8% versus 2.8%), and vegetables (0.5% versus 1.0%). Individuals who reported not using sugar or AS presented a higher percentage contribution to the daily energy intake for fruits (6.2%) than those who used sugar, AS, or both (Table 3).

## 4. Discussion

The use of sugar to sweeten foods and beverages is highly widespread in Brazil, and was more commonly practiced by men, adolescents, residents of rural areas, those who live in the northern and northeastern regions, and among low-income individuals. On the other hand, the use of AS, either exclusively or in combination with sugar, was reported by approximately 13% of the population, and was more frequent among elderly, women, overweight individuals, those living in urban areas, in the south and southeast, and among high-income individuals compared with their counterparts. It was noteworthy that the use of AS among the elderly was 10 times higher than that of the adolescents, and almost triple that of adults; additionally, over a fifth of Brazilians in the highest income quartile reported the addition of AS to foods and beverages. Apparently, the use of AS was related to lower energy intake, since the average energy intake was lower among those using AS only in comparison with those using sugar exclusively or in combination with AS, except for the midwest region. In average, the use of sugar to sweeten foods and beverages implies an increase of 186 kcal daily, corresponding to a 10% increase in the total energy intake.

The most important differences in the profile of food group consumption according to the type of sweetener were concerning for sugar-sweetened beverages, snacks and chips, sweets and desserts, beans, vegetables, and fruits. Sugar use was related to the Brazilian traditional eating pattern, including rice, beans, and coffee [24,25], whereas the use of AS was related to a mixed eating pattern, including salty snacks and fruits and vegetables. Comparing individuals who reported the exclusive use of sugar and those who only used AS, the consumption of sugar-sweetened beverages, sweets and desserts, and beans was greater, while the consumption of snacks and chips, vegetables, and fruits was smaller.

Consistently with our findings, a study analyzing data from the Household Budget Survey showed that, in Brazil, three-fourths of the calories were from added sugars such as table sugar and other caloric sweeteners [26]. Furthermore, the high intake of table sugar among men and adolescents is consistent with the high proportion of unfavorable eating habits observed in these groups of population [27,28,29].

The proportion of Brazilians who sweeten foods and beverages with AS was similar to the observed proportion of American adults (≥18 years) who use low-caloric sweetener packets (14.1%), as investigated in the 2009–2010 and 2011–2012 National Health and Nutrition Examination Surveys [30]. Additionally, the results of this study are also consistent with several other studies observing that AS consumption is more frequent among women [31,32,33,34], elderly [15,30,31,32], overweight [30,31,32,33,34,35,36], and highly educated individuals [31,32,34,36], and those in the higher socioeconomic strata [30,31,36]. Moreover, in the Longitudinal Study of Adult Health (ELSA)-Brazil cohort, the “diet/light” eating pattern was more common among women, elderly, and highly educated individuals [37].

In the United States, a substantial increase in the purchase and consumption of artificially sweetened foods and beverages has been verified in recent decades [2,13,14]. AS consumers are more likely to engage in dieting behavior [31,38], which is characterized by a lower daily energy intake; a lower contribution of carbohydrates, sugar, and sugar-sweetened beverages to the total energy intake; and higher overall dietary quality [31,32,34,38]. Consistently, the users of AS in our study presented a higher contribution of fruits and vegetables to the total energy intake, and had lower means of total energy intake.

AS were conceptualized as an alternative sweetener for diabetic individuals; later, they were popularized as a strategy to reduce caloric content and promote weight loss [10]. Despite that, the number of randomized controlled trials and observational studies that have reported potential associations between the consumption of AS and artificially sweetened beverages and weight gain and higher adiposity [31,36,39], greater cardiovascular risk [35], negative changes in intestinal microbiota, glucose intolerance, and greater risk of diabetes mellitus [33,40,41,42] has been continuously increasing. Nonetheless, the metabolic and clinical effects of using AS remained unclear [20].

Several intervention strategies and nutrition policies have focused on reducing the levels of sugar consumption in the population [43,44], which possibly leads to replacing table sugar with AS. Even so, considering the harmful effects of AS, healthy eating promotion strategies should also aim to limit their consumption.

Despite the possible limitations in this study, our data are consistent with those of other studies that investigated table sugar or AS consumption in Brazil [15,26]. A limitation is the possibility of underreporting by the use of food records. Applying food records can help individuals by not requiring them to rely on their memory to report foods consumed, and provide more accurate information regarding the quantities consumed [45]; in this study, various strategies to reduce measurement errors were applied, such as continuous interviewers’ training and supervising, and the use of a written manual for the participants. Although food consumption underreporting was not evaluated in the present analysis, a validation study on the dietary assessment method adopted in the Brazilian National Dietary Survey using doubly labeled water indicated an energy intake underestimation of approximately 30% [46]. Furthermore, the use of the first day of food records may be pointed to as a limitation of this study. Nevertheless, it is recognized that single 24-h recalls and food records provide reliable estimates for population means in extent studies. Additionally, the first day of food record usually afford more complete data than the following days [47].

This investigation is unique, because it helps characterize the type of sweetener added by Brazilians in their beverages and foods. This is the first nationally representative study that evaluates the type of sweetener that is usually added in beverages and foods and their contribution to daily energy intake, as well as the relationship between the type of sweetener used and the most commonly consumed foods. Additionally, this study used a nationwide representative sample that is capable of discriminating the estimates at geographic regions and income levels in Brazil. Moreover, this study sets the basis to monitor sweeteners’ use in this country, allowing future investigations on their impact on diet quality and health and nutrition indicators, as well as verifying whether a possible migration from table sugar to AS occurs in the national setting.

## 5. Conclusions

In summary, this study showed that approximately 20% of the elderly, and individuals in the highest quartile of income, were the greatest consumers of AS. The use of AS is associated with lower energy intake, and is commonly observed in individuals who consumed more fruits and vegetables, which may indicate that a health-conscious population subgroup chooses AS.

## Figures and Tables

**Table 1:** Sample size and weighted prevalence of sweetener use. Brazil, National Dietary Survey 2008–2009.

Characteristics	Sample Size (%)	Prevalence of Use (% (95% CI)) *			
		Sugar	Artificial Sweeteners	Sugar and Artificial Sweeteners	Neither Sugar nor Artificial Sweetener
Total (*n =* 32,749)	-	85.7 (84.7; 86.6)	7.6 (6.9; 8.3)	5.1 (4.6; 5.8)	1.6 (1.3; 1.9)
Age group					
Adolescents	21.6	94.9 (93.9; 95.8)	1.9 (1.3; 2.6)	1.9 (1.4; 2.4)	1.3 (0.9; 2.0)
Adults	64.6	86.1 (85.0; 87.1)	6.9 (6.2; 7.7)	5.5 (4.8; 6.2)	1.5 (1.2; 1.9)
Elderly	13.8	69.3 (66.3; 72.2)	19.9 (17.6; 22.4)	8.7 (6.8; 11.1)	2.0 (1.4; 3.0)
Sex					
Female	49.9	82.0 (80.7; 83.2)	9.7 (8.8; 10.6)	6.5 (5.8; 7.3)	1.9 (1.5; 2.3)
Male	50.1	89.4 (88.4; 90.4)	5.5 (4.8; 6.4)	3.8 (3.2; 4.4)	1.3 (0.9; 1.7)
Weight status					
No excess weight	58.2	88.5 (87.4; 89.5)	5.9 (5.1; 6.7)	4.3 (3.7; 4.9)	1.4 (1.1; 1.8)
Overweight or obese **	41.8	81.8 (80.4; 83.1)	10.0 (9.0; 11.1)	6.4 (5.6; 7.3)	1.8 (1.4; 2.3)
Place of domicile					
Urban	83.6	84.2 (83.1; 85.3)	8.5 (7.7; 9.4)	5.6 (5.0; 6.3)	1.7 (1.4; 2.0)
Rural	16.4	93.3 (91.5; 94.7)	3.0 (2.4; 3.6)	2.8 (1.7; 4.5)	0.9 (0.4; 1.9)
Regions of Brazil					
North	7.5	91.7 (89.8; 93.3)	2.9 (2.2; 3.7)	3.7 (2.7; 5.2)	1.7 (1.1; 2.6)
Northeast	27.3	90.6 (89.5; 91.6)	5.8 (5.1; 6.6)	3.1 (2.6; 3.7)	0.5 (0.3; 0.7)
Southeast	43.2	82.7 (80.8; 84.5)	9.5 (8.2; 10.9)	6.3 (5.3; 7.5)	1.5 (1.1; 2.2)
South	14.8	81.2 (78.5; 83.5)	9.5 (7.8; 11.4)	6.0 (4.5; 7.8)	3.4 (2.6; 4.5)
Midwest	7.2	87.9 (85.2; 90.1)	4.5 (3.3; 6.1)	5.8 (4.6; 7.4)	1.8 (1.0; 3.2)
Quartile of income					
1st	24.3	96.0 (94.9; 96.8)	1.6 (1.1; 2.2)	1.3 (0.9; 1.8)	1.2 (0.6; 2.1)
2nd	25.0	92.9 (91.7; 93.9)	3.5 (2.8; 4.8)	2.5 (1.9; 3.1)	1.2 (0.8; 1.9)
3rd	25.3	86.7 (85.1; 88.2)	7.4 (6.3; 8.6)	4.8 (3.9; 6.0)	1.1 (0.7; 1.6)
4th	25.4	74.1 (71.9; 76.2)	14.0 (12.5; 15.8)	9.4 (8.2; 10.9)	2.4 (1.8; 3.0)

* CI 95%—Confidence Intervals 95%. ** Overweight or obese: Adolescents—*z*-scores of BMI above +1 of the reference distribution; Adults—BMI ≥ 25kg/m^2^; Elderly—BMI ≥ 27 kg/m^2^. BMI: body mass index. 1st, 2nd, 3rd, 4th: the first, second, third and fourth quartiles of income.

**Table 2:** Daily energy intake according to the type of sweetener. Brazil, National Dietary Survey 2008–2009.

Characteristics	Total Daily Intake of Energy (kcal)			
	Sugar	Artificial Sweeteners	Sugar and Artificial Sweeteners	Neither Sugar nor Artificial Sweetener
Total	1955 ^a^	1636 ^b^	1832 ^c^	1770 ^b,c^
Sex				
Female	1740 ^a^	1530 ^b^	1724 ^a,^	1514 ^c^
Male	2154 ^a^	1821 ^b^	2020 ^a^	2150 ^a^
Age group				
Adolescents	2066 ^a^	1750 ^b^	2029 ^a,b^	2014 ^a,b^
Adults	1963 ^a^	1676 ^b,c,d^	1872 ^a^	1815 ^a,c,d^
Elderly	1672 ^a^	1554 ^b,c,d^	1649 ^a,c^	1360 ^c^
Weight status				
No excess of overweight	1954 ^a^	1624 ^b,d^	1810 ^c,e^	1783 ^d,e^
Overweight or obese (BMI ≥ 25 kg/m^2^)	1957 ^a^	1646 ^b,c^	1853 ^a^	1756 ^a,c^
Place of domicile				
Urban	1956 ^a^	1635 ^b,e^	1824 ^c^	1789 ^c,d,e^
Rural	1955 ^a^	1652 ^b^	1919 ^a^	1589 ^b^
Regions of Brazil				
North	2148 ^a^	1811 ^b^	2088 ^a,b^	1805 ^b^
Northeast	1916 ^a^	1602 ^b,c^	1929 ^a^	1524 ^c^
Southeast	1954 ^a^	1636 ^b^	1803 ^c,d^	1923 ^a,d^
South	1963 ^a^	1587 ^b^	1808 ^b^	1556 ^b,c^
Midwest	1897 ^a^	1901 ^a^	1706 ^b^	2038 ^a^
Quartile of income				
1st	1805 ^a^	1474 ^b^	1815 ^a^	1676 ^a,b^
2nd	1945 ^a^	1707 ^b^	1741 ^b^	1570 ^b^
3rd	1969 ^a^	1538 ^b,d^	1812 ^c,e^	1671 ^d,e^
4th	2063 ^a^	1676 ^b^	1858 ^c,d^	1902 ^a,d^

Estimates obtained by General Linear Models with Bonferroni correction. Different letters (a, b, c, d, e) indicate significant differences between the estimates for each category (type of sweetener) on the row (*p*-value < 0.05).

**Table 3:** Contribution (%) to the daily intake of energy of the food groups according to the type of sweetener. Brazil, National Dietary Survey, 2008–2009.

Food groups	Percentage Contribution to Daily Energy Intake *			
	Sugar	Artificial Sweeteners	Sugar and Artificial Sweeteners	None of These Products
Eggs	1.5 ^a^	0.7 ^b^	0.9 ^b^	1.1 ^a,b^
Sugar-sweetened beverages	2.3 ^a^	1.6 ^b,c^	2.0 ^a,c^	2.9 ^b^
Snacks and chips	1.8 ^a^	2.8 ^b^	1.6 ^a^	2.9 ^b^
Beans	10.7 ^a^	7.8 ^b^	8.2 ^b^	8.4 ^b^
Pasta	3.6 ^a^	3.6 ^a^	3.8 ^a^	4.6 ^a^
Fruit juice	4.8 ^a^	3.5 ^b^	5.4 ^a^	2.9 ^b^
Butter or margarine	2.4 ^a^	2.4 ^a^	2.2 ^a^	2.7 ^a^
Vegetables	0.5 ^a^	1.0 ^b^	0.9 ^b^	0.6 ^a^
Fruits	2.7 ^a^	5.5 ^b^	3.9 ^c^	6.2 ^d^
Cheese	0.9 ^a^	2.5 ^b^	1.7 ^c^	1.3 ^a^
Processed meats	1.9 ^a^	1.7 ^a^	2.2 ^a^	1.4 ^a^
Poultry	4.7 ^a^	6.2 ^b^	4.7 ^a^	4.2 ^a^
Breads	8.6 ^a^	10.8 ^b^	8.8 ^a^	9.7 ^a^
Rice	14.9 ^a^	12.2 ^b^	12.8 ^b,c^	14.2 ^a^
Meat	10.2 ^a^	9.9 ^a^	10.3 ^a^	9.1 ^a^
Sweets and desserts	5.0 ^a^	4.0 ^b^	5.9 ^a^	4.8 ^a^
Coffee and tea	6.9 ^a^	2.6 ^b,e^	4.7 ^c^	2.8 ^d,e^
Candies and Chocolate	1.1 ^a^	1.3 ^a^	1.6 ^a^	1.5 ^a^
Roots and tubers	3.9 ^a^	3.3 ^b^	2.8 ^b^	2.9 ^b^
Milk	2.3 ^a^	3.0 ^b,c^	2.6 ^a,c^	3.1 ^a,b^

Estimates obtained by general linear models with correction for Bonferroni. Different letters (a, b, c, d, e) indicate significant differences between the estimates for each category (type of sweetener) on the row (*p*-value <0.05). * Contribution to daily energy intake = (food group energy × 100)/total energy intake.
